# *Ehrlichia* Prevalence in *Amblyomma americanum*, Central Texas

**DOI:** 10.3201/eid1007.030792

**Published:** 2004-07

**Authors:** Scott Wesley Long, J. Mathews Pound, Xue-jie Yu

**Affiliations:** * University of Texas Medical Branch, Galveston, Texas, USA;; † United States Department of Agriculture – Agricultural Research Service, Kerrville, Texas, USA

**Keywords:** letter, *Ehrlichia ewingii*, *Ehrlichia chaffeensis*, *Amblyomma americanum*, Texas epidemiology

*Suggested citation for this article*: Long SW, Pound JM, Yu X. *Ehrlichia* prevalence in *Amblyomma Ehrlichia chaffeensis* and *E. ewingii*, agents of human monocytic ehrlichiosis and ehrlichiosis ewingii, respectively, are transmitted by the lone star tick, *Amblyomma americanum*, which is found from west-central Texas northward to Iowa, and southeastward to the Atlantic Coast (1). In *A. americanum*, *E. chaffeensis* has been found in several states, while *E. ewingii* has only been found in North Carolina, Florida, and Missouri (1,2). *E. ewingii* infection in white-tailed deer (*Odocoileus virginianus*), a potential reservoir, has been found in the states mentioned previously as well as in Kentucky, Georgia, and South Carolina (3,4).

Human ehrlichioses are underdiagnosed in the United States and may be as prevalent as Rocky Mountain spotted fever in some areas (1). Ehrlichioses are prevalent in Texas, and fatal cases have been reported (1,5). This study was conducted to examine ticks from central Texas for *Ehrlichia* and provide information to increase public health awareness of this problem. Adult *A. americanum* ticks were collected from a 38.8-hectare game fenced-pasture (Plot #8) in the Kerr Wildlife Management Area, Kerr County, Texas. Ticks were trapped by using blocks (approximately 85 g) of dry ice centered on smooth, white, nylon cloths measuring approximately 1 m^2^. These traps were placed on the ground in the brush or in areas under tree canopies for approximately 1 h.

Trapped adult *A. americanum* were frozen in liquid nitrogen and then bisected with a sterile scalpel. Halves of the bisected ticks were stored at –80ºC. The other halves were pooled in groups of six. DNA was extracted from these pools by using the QIAamp DNA Mini Kit (Qiagen Inc., Valencia, CA), and evaluated by using a nested species-specific 16S rRNA gene polymerase chain reaction (PCR) for *E. chaffeensis* and *E. ewingii*, with *E. canis* as a negative control. The first-round primers were genus-specific for *Ehrlichia* (ECC and ECB). The forward primers of the nested PCR were HE1, EE72, and Ecan, which were specific to *E. chaffeensis*, *E. ewingii*, and *E. canis*, respectively. The reverse primer is a common primer (HE3) for all species (6–8). An aliquot of the negative control reaction containing no DNA template was carried through both rounds of the nested PCR with every reaction set. A dilution series of stock *E. chaffeensis* DNA mixed with tick DNA showed no substantial inhibition of the PCR, even with DNA concentrations as low as 0.2 ng/mL.

Tick pools positive for *E. chaffeensis* or *E. ewingii* by PCR were examined by using DNA from the individual tick halves. DNA was extracted by using the Nucleobond DNA/RNA Isolation Kit (BD Biosciences Clontech, Palo Alto, CA). To confirm positive PCR results for individual ticks, first-round amplicons (primers ECB and ECC) were separated by electrophoresis. The 478-bp band was recovered using the QIAquick Gel Extraction Kit, then cloned into the pCR2.1-TOPO vector with the TOPO TA cloning kit (Invitrogen, Carlsbad, CA). DNA sequences were obtained from both directions of the insert in the recombinant plasmids by using PE Applied Biosystems (Foster City, CA) 373XL automated DNA sequencers in the UTMB Sequencing Core.

Of the 66 adult *A. americanum* ticks examined, 5 were positive for *E. ewingii* (7.6%). The 16S rRNA gene sequences from these five positive samples were most similar to the *E. ewingii* 16S rRNA gene sequence (GenBank accession no.U96436). Sequence variations are summarized in the Table. These mutations may result from polymerase errors prior to cloning. *E. ewingii* has never been cultured or handled by our laboratory, and all negative controls for the nested PCR were negative, minimizing the possibility of false-positive results.

**Table:** Sequence variation of the 16S rRNA gene in *Ehrlichia ewingii* detected in *Amblyomma americanum* ticks from central Texas, compared to the partial 16S rRNA gene sequence of *Ehrlichia ewingii* in Genbank (accession U96436)^a^

	G16	A93G	A157G	T190C	A429G	C474
Tick B5	–	–	–	–	–	–
Tick B7	+	–	–	+	–	–
Tick D1	–	+	+	–	+	+
Tick D2	–	+	+	–	–	–
Tick D4	–	+	+	–	–	–

^a^+, mutation present, –, mutation not present.

This is the first report of ticks infected with *E. ewingii* in states other than North Carolina, Florida, or Missouri. Ticks are found in damp wooded areas (1,9). Seasonal population changes have been associated with climatic factors, including precipitation, temperature, and day length (9–11). These ticks were collected during August, one of the hottest months of the year in Texas, with temperatures averaging 33°C. Adult ticks are more abundant earlier in the summer, and the actual prevalence of *E. ewingii* infection may be higher. August is a dry month in Texas, averaging 2.32 inches of an annual rainfall of 26 to 30 inches (12). Although cases of *E. ewingii* infection have not been reported from Texas, this study shows the presence of ticks infected with *E. ewingii* in Texas.

No ticks infected with *E. chaffeensis* were found in this sample. The prevalence of *E. chaffeensis* may be so low that it was not detected in the small sample size. Also, *E. chaffeensis* may not survive well at this extreme of the host range. Infection exclusion may occur in the tick or reservoir hosts (or both), such that an established population of one ehrlichial species prevents another ehrlichial species from establishing itself. This phenomenon has been noted in the related rickettsial organisms *Rickettsia peacockii* and *R. rickettsii* in the Rocky Mountain wood tick, *Dermacentor andersoni* (13).

Another finding involves using nested 16S rRNA PCR to identify ehrlichial infection. These primers are not as specific as thought previously. Arthropods should be carefully cleaned to prevent contamination by *Shigella* and other soil contaminants. A single positive-nested PCR reaction should not be considered sufficient for positive identification of the organism. Sequencing of the outer PCR product, or another confirming method, should be used to positively identify the organism. Primers directed to more divergent sequences, such as the *dsb* gene, should be utilized in place of, or in addition to, 16S rRNA gene PCR (14).

This study was supported by a fellowship for Scott W. Long from the Sealy Center for Vaccine Development, University of Texas Medical Branch, and a grant from the National Institute of Allergy and Infectious Diseases (AI45871).
